# Focus on One or More? Cultural Similarities and Differences in How Parents Talk About Social Events to Preschool Children

**DOI:** 10.3389/fpsyg.2021.778960

**Published:** 2022-01-04

**Authors:** Megumi Kuwabara, Linda B. Smith

**Affiliations:** ^1^Child Development Department, California State University, Dominguez Hills, Carson, CA, United States; ^2^Department of Psychological and Brain Sciences, Indiana University, Bloomington, IN, United States

**Keywords:** cross-cultural, social events, parent’s talk, children, puppet shows

## Abstract

How parents talk about social events shapes their children’s understanding of the social world and themselves. In this study, we show that parents in a society that more strongly values individualism (the United States) and one that more strongly values collectivism (Japan) differ in how they talk about negative social events, but not positive ones. An animal puppet show presented positive social events (e.g., giving a gift) and negative social events (e.g., knocking over another puppet’s block tower). All shows contained two puppets, an actor and a recipient of the event. We asked parents to talk to their 3- and 4-years old children about these events. A total of 26 parent–child dyads from the United States (*M* = 41.92 months) and Japan (*M* = 42.77 months) participated. The principal dependent measure was how much parent talk referred to the actor of each type of social event. There were no cultural differences observed in positive events – both the United States and Japanese parents discussed actors more than recipients. However, there were cultural differences observed in negative events – the United States parents talked mostly about the actor but Japanese parents talked equally about the actor and the recipient of the event. The potential influences of these differences on early cognitive and social development are discussed.

## Introduction

The world of children is filled with both positive and negative social events. A child may help another child who is having difficulty opening a door or a child may grab a toy away from another child. These events provide learning opportunities for children about prosocial and antisocial events. The extant evidence strongly indicates that how parents talk about these positive and negative social events play a critical role in children’s emerging interpretations of these events (Brownell et al., 2013; Spinrad and Gal, 2018), the development of social values (e.g., Walker and Taylor, 1991; Eisenberg et al., 2001), children’s moral development (e.g., Laible, 2004; Recchia et al., 2014), emotion regulation (e.g., Eisenberg et al., 2001), social/emotional understanding (e.g., Laible, 2004), prosocial behaviors (e.g., Garner et al., 2008), self-esteem (e.g., Reese et al., 2007), and self-concept (e.g., Welch-Ross et al., 1999). Considerable research implicates cultural differences in the development and interpretation of prosocial and antisocial events (Fu et al., 2007; Chiu Loke et al., 2014; Blake et al., 2015; Cowell et al., 2017; Callaghan and Corbit, 2018).

The context for the current study is the widely documented East–West cultural differences in the conceptualization of the roles of an individual (see Markus and Kitayama, 1991; Henrich et al., 2010 for review). Western cultures are characterized as valuing an individual whereas Eastern cultures are characterized as valuing harmony and community. These differences have been linked to differences in parenting practices and parent expectations about their children (see Bornstein, 2012 for review). For example, in individualized Western societies, parents view their children as separate entities and emphasize autonomy and independence whereas parents from collectivistic Eastern societies view their children as extensions of themselves and emphasize a feeling of interconnectedness (Rothbaum et al., 2000). Several studies have shown that school-age children and adults in Eastern and Western cultures differ in responses to and interpretation of prosocial and antisocial behaviors (Fu et al., 2007; Heyman et al., 2011; Chiu Loke et al., 2014), and other studies indicate that these differences are also evident in much younger children (Taylor et al., 2002; Kuwabara et al., 2011; Shimizu et al., 2021). Several studies suggest that these cultural differences may be most pronounced for the antisocial behaviors of others (Fu et al., 2007; Chiu Loke et al., 2014). For example, Wang and Fivush (2005) found that parents from Eastern and Western cultures differed in conversations about two past events – positive (e.g., family vacation) and stressful (e.g., child illness) events. They found no differences in the talk about positive events but reliable differences in talking about stressful events. Chinese parents focused more on the interpersonal contexts of feelings (e.g., you were sad because I was scolding you) whereas the United States parents focused on non-social contexts and causes (e.g., you were sad because you were too sick to attend an event). United States parents described the events or objects in the environment (e.g., having a surgery) as a cause of their child’s negative emotion whereas Chinese parents describe the interpersonal situations (e.g., parents scolding the child) as a cause of their child’s negative emotion. Here, we focused on the potential role of parent talk to preschool children about positive and negative social events to understand the potential cultural similarities and differences in interpretations of prosocial and antisocial behaviors.

Previous studies indicate Eastern and Western cultural differences in how visual attention is distributed to people in a scene (e.g., Masuda et al., 2008), with adults from Eastern cultures distributing attention across people and adults from Western cultures focusing on one. These differences have also been documented in non-social attentional tasks with adults and children (Nisbett and Miyamoto, 2005; Duffy et al., 2009; Moriguchi et al., 2012; Imada et al., 2013; Senzaki et al., 2014; Kuwabara and Smith, 2016; Köster and Kärtner, 2018) and have been suggested to derive originally from differences in more collective versus more individualized social behaviors (Markus and Kitayama, 1991; Nisbett, 2003). These cultural differences in the distribution of visual attention suggest that parents in Eastern cultures may talk about *both* the actor and recipient of social events. Alternatively, given that cultural differences may be greater with negative social events, parent talk about negative social events may differ more across cultures with respect to whether the feelings and behaviors of all participants in the interaction are considered.

In sum, the study was designed to determine how parent talk focuses attention on an actor and the recipient of a social event – attention to just one or both – when talking about positive and negative social events. Overall, the expectation is that United States parents may talk about just one individual, the actor, and Japanese parents may talk about both. However, Wang and Fivush’s (2005) findings may indicate that the expected cultural differences may be most measurable in the talk about negative social events. The study thus measures two factors suggested by prior work of East–West cultural differences: interpretations of positive and negative social events and the distribution of attention to a single character or all characters. The focus is on parent talk to preschool children because this is a period in which parent talk about emotional events are known to influence the development of moral and social reasoning (Brownell et al., 2013; Spinrad and Gal, 2018) and an age at which cross-cultural differences begin to become apparent (Kuwabara et al., 2011; Shimizu et al., 2021). Our approach follows a widely used method in the studies of older children’s interpretations of prosocial and antisocial behaviors: we present vignettes in which characters act out these behaviors (Fu et al., 2007; Heyman et al., 2011; Chiu Loke et al., 2014). In our case, the vignettes are silent puppet shows and we asked the parents to narrate to the child what was happening.

## Materials and Methods

### Participants

The parents are the subject of this experiment: 29 parents with their child from the United States and 27 parents with their child from Japan participated in the study. Using the effect size found in Wang and Fivush’s (2005) study, power analysis indicates that 38 participants (19 participants from each country) would be sufficient to detect a medium effect with power set at 0.80 and α = 0.05. Three dyads from the United States and one dyad from Japan were excluded from this study due to failures of the recording system. Therefore, 26 parents (24 mothers and two fathers) from each country were included for analysis. The mean age of the United States children (14 males and 12 females) was 41.92 months and the mean age of Japanese children (12 males and 14 females) was 42.77 months, across countries the children ranged in age from 36 to 52 months. All children were monolingual speakers (the United States children were monolingual English speakers and Japanese children were monolingual Japanese speakers). Recruitment and testing procedures in both countries were approved by the Indiana University Institutional Review Board.

### Stimuli

**FIGURE 1:**
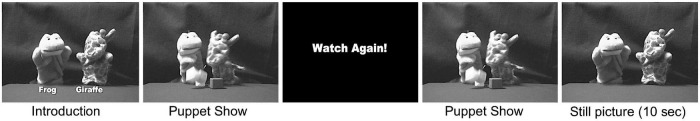
The Sequence of a Puppet Show. The sequence of a puppet show from the introduction of the characters to the still picture (10 s). Each show was presented twice in immediate succession with the “Watch Again!” slide as a cue for the second presentation.

We created 14 puppet shows containing seven different animal puppets (lion, cat, monkey, pig, giraffe, dog, and rabbit) to avoid facial expressions. Seven shows were filler shows and involved neutral events (e.g., bunny eating green peas) that included one puppet or joint actions of two or three puppets. These filler shows were included to reduce the obviousness that the experiment was principally about positive versus negative social events. The seven experimental shows included interactions among two puppets in which one puppet was the actor and the other puppet was the recipient of the action (see Appendix A). Each puppet show ranged from 11 to 20 s. Before each show began, the characters were introduced and named, so parents could use the correct names during the conversation. Each show was presented twice in immediate succession. At the end of the second showing, 10 s still pictures of characters were included to assist parents in finishing their talk about the events before the start of the next show (see Figure 1 for the sequence of a show). These 14 shows were combined to create a video of puppet shows in two randomized orders, which were randomly assigned to each participant. The total duration of 14 puppet shows was 10 min and 10 s.

Our analysis focused on seven experimental scenes (see Appendix A for scene description) that contained two puppets, an actor and a recipient of the scene. There were three positive scenes (giving a present, sharing ice cream, and being gentle) and four negative scenes (knocking down the tower of blocks that were built by another puppet, stealing a ball from another puppet, hitting another puppet with a bag, and scaring another puppet). Each of these scenes contained an actor and a recipient of the action. For example, one of the scenes included a lion giving a gift to a frog. For this scene, the lion was coded as an actor because he was a giver of the gift and the frog was coded as a recipient of the action (the receiver of the gift).

### Procedure

Each dyad watched these shows without sound in a quiet room with a laptop computer. Parents were told that these puppet shows display events of the kinds that children often experience. They were asked to talk to their child about these events as they normally would at home. The puppet shows included no sounds because the goal of the study was to elicit potentially different biases of interpretation in the two cultures without disruptions and we did not want to influence parents’ narration beyond the visual behaviors.

### Coding

Each session was transcribed by native speakers. The dependent measure was parent talk. Talk by children was very sparse, and most said very little. From the transcriptions of parent talk, we counted which puppet, actor or recipient, was mentioned by each parent (explicit naming) as well as the total number of words that parents characterized each puppet positively (e.g., was kind) or negatively (e.g., not nice to do that). Two coders coded for the explicit naming of characters for 50% of dyads (26 dyads) and their reliability was 100% agreement, *r* = 1.0.

The Japanese language allows speakers to drop the subject and/or object of sentences, but English does not. For example, *kaeru ga tsumiki wo tsundeiru* (frog is building a block tower) can be expressed *kaeru ga tsundeiru* (frog is building), *tsumiki wo tsundeiru* (is building a block tower), *tsundeiru* (building) in Japanese, but not English. To account for language differences, we also coded which character was referred to based on verbs or adjectives of each utterance. For example, if a parent said, “[frog] is building a block tower,” this was counted as “frog” because, in the scene, the frog was the one building a block tower. If a parent said, “that was nice,” we counted as “lion” because, in the scene, the lion was being nice giving a present to the frog. Two coders coded for the character reference based on verbs or adjectives for 50% of dyads (26 dyads) and their reliability was high, *r* = 0.96. For each puppet show, the sum of the explicit mention of character names and character references based on verbs or adjectives was calculated for actor and recipient. Each adjective or verb used to describe the puppets was also coded for the valence of the word – positive (e.g., good, nice, and like) or negative (e.g., not nice, mean, and does not like).

## Results

The number of character references for 14 puppet shows differed across individuals in both cultures; the number of references in the United States ranged from 23 to 169; in Japan from 22 to 170 references. For both countries, there was no difference in the total number of the actor and recipient references between positive and negative scenes, *t*(25) = 0.02, *p* = 0.98, *d* = 0.004 for the United States and *t*(25) = −1.83, *p* = 0.08, *d* = 0.36 for Japan. Because the main hypotheses are about the balance of a different kind of talk about the actor versus recipient puppet, the main dependent variable in the analyses was the proportion of the talk about the actor (calculated by the total number of actor references divided by the total number of references of actor and recipient). This score was calculated for positive scenes and negative scenes for each participant. The proportion of the actor talk (see Table 1) was entered into an rANOVA for 2 scene types (positive versus negative) × 2 countries (the United States versus Japan) design, which yields the significant interaction between scene types and country, *F*(1,50) = 8.84, *p* < 0.01, η^2^ = 0.15, significant main effect of scene type, *F*(1,50) = 29.40, *p* < 0.01, η^2^ = 0.37, but no significant main effect of country, *F*(1,50) = 1.46, *p* = 0.23, η^2^ = 0.03. To further examine the interaction, the amount of parent talk about the actor for positive versus negative scenes was entered into paired sample *t*-tests (with *p*-value sets at 0.01 for multiple comparisons). These comparisons showed that the only significant difference was found in Japanese parents, *t*(25) = 5.96, *p* < 0.01, *d* = 1.17, but not in the United States parents, *t*(25) = 1.73, *p* = 0.10, *d* = 0.34. Further analyses to see whether the actor talk was greater than 0.50, which indicates biased emphasis to talk about actors more than recipients of scenes, the actor talk for each scene type for each country was entered into separate one-sample *t*-tests (with *p*-value set at 0.01 for multiple comparisons). By these analyses, United States parents were biased to talk about the actor in both positive, *t*(25) = 4.87, *p* < 0.01, *d* = 0.96 and negative scenes, *t*(25) = 4.18, *p* < 0.01, *d* = 0.82 as were Japanese parents talking about positive scenes, *t*(25) = 11.06, *p* < 0.001, *d* = 2.17, but not negative scenes, *t*(25) = −0.57, *p* = 0.57, *d* = 0.11, which showed balanced talk of the actor and the recipient.

**TABLE 1:** Proportion of the actor talk.

Scene type	United States		Japan	
	*M*	SD	*M*	SD
Positive scenes	0.64	0.15	0.68	0.08
Negative scenes	0.58	0.10	0.48	0.14

*The mean (M) and standard deviation (SD) of the proportion of the actor talk for positive and negative scenes for United States parents and Japanese parents.*

To examine what kinds of words were used to describe positive and negative scenes and whether they differ by country, the valence – positive (e.g., good, nice, and like) or negative (e.g., not nice, mean, and does not like) of each adjective or verb used to describe the puppets was converted to the proportion to account for individual differences in the number of puppet references. The proportion of positive valence words were entered into 2 scene types (positive versus negative) × 2 countries (the United States versus Japan) rANOVA, which yield a significant main effect of scene type, *F*(1,43) = 269.80, *p* < 0.001, η^2^ = 0.86, but no significant main effect of the country, *F*(1,43) = 0.02, *p* = 0.90, η^2^ = 0.00 or interaction, *F*(1,43) = 0.25, *p* = 0.62, η^2^ = 0.006 which shows that there were no cultural differences in what kinds of words were used to describe the puppets. During the positive events, almost all of the adjectives and verbs used by both parents (the United States and Japanese) had positive valence (*M* = 0.97, SD = 0.08 for the United States and *M* = 0.99, SD = 0.03 for Japanese) and during the negative events, majorities of adjectives and verbs used by both parents (the United States and Japanese) had negative valence (*M* = 0.73, SD = 0.27 for the United States and *M* = 0.75, SD = 0.32 for Japanese).

## Discussion

We examined whether there are cultural differences in how parents discuss social events with their children and in particular if there were cultural differences in the distribution of attention to one character or both characters in the social interaction and whether cultural differences were more pronounced in discussions of negative social events. As predicted, there were no cultural differences in positive scenes – both the United States and Japanese parents discussed actors more than recipients of the scenes. The cultural difference was primarily observed in the negative scenes – the United States parents discussed actors, the character who exhibited the negative social behavior, more than the recipient of the bad behavior, but Japanese parents discussed both characters equally. Our results are similar to the results from the Wang and Fivush (2005) study that cultural differences in how parents talk to their children are more exaggerated in negative social events. Potentially, cultural differences may more generally be pronounced in the interpretation of antisocial events than the prosocial events (e.g., Miller, 1986), which might influence other social developments (e.g., Eisenberg et al., 2001; Laible, 2004; Reese et al., 2007; Garner et al., 2008) and might contribute to different developmental trajectories. These differences in how parents talk to their children could also be one of the potential transmission vectors of cultural differences in attention and perception that have been observed in children and adults – individuals from Western cultures attending to one focal object or person versus individuals from Eastern cultures attending to multiple objects or people in scenes (Nisbett and Miyamoto, 2005). The results of negative social events in this study align well with the well documented (Nisbett and Miyamoto, 2005; Masuda et al., 2008; Duffy et al., 2009; Moriguchi et al., 2012; Imada et al., 2013; Senzaki et al., 2014; Kuwabara and Smith, 2016; Köster and Kärtner, 2018) of more distributed versus more focal visual attention to social and non-social stimuli for Eastern versus Western participants. Parent talk in emotionally laden social contexts that directs attention to all or only the focal character may play a role in the formation of these cultural differences.

**FIGURE 2:**
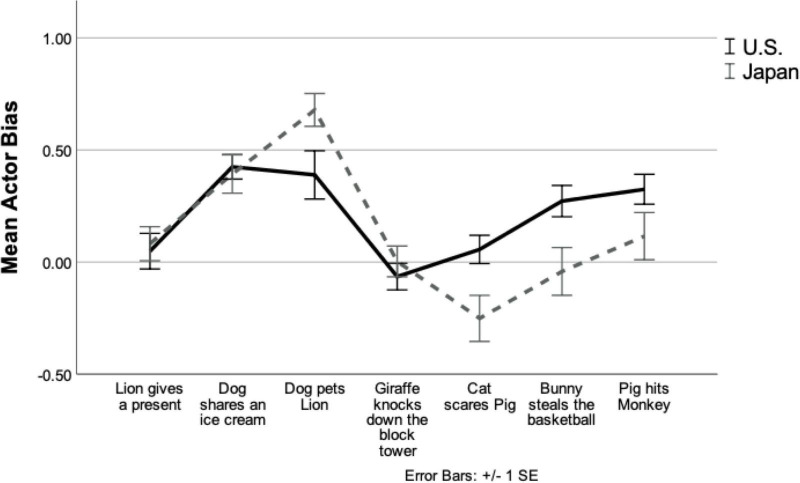
Line graph of mean actor bias of each puppet show. The mean actor bias (the proportion of the actor talk over the proportion of the recipient talk) is shown for each show. The positive number indicates more reference was given to the actor of the scene, the negative number indicates more reference was given to the recipient of the scene, and zero indicates both characters were discussed equally. The United States results are displayed in a solid black line and Japanese results are displayed in a dotted gray line. The first three scenes are positive social events and the last four scenes are negative social events. Error bars were set as ±1 SE.

Figure 2 shows the mean bias in the two cultures to talk about the actor. The observed differences between cultures are clearly not all or none. For example, the scene depicting the dog petting the baby lion seems to show cultural differences in the degree to which talk is biased to the actor in the scenario even though this is a positive scene whereas other positive scenes did not elicit cultural differences. The scene depicting the giraffe knocking over the block tower that was built by the frog did not show any cultural difference even though this is a negative scene whereas other negative scenes elicit cultural differences. These effects of item type, which might be due to the intensity of positivity and negativity present a potential path to understanding why negative events typically lead to stronger cultural differences. Studying similarities and differences across cultures in how parents talk about discrete emotional events (e.g., anger and fear) rather than focusing on general positive and negative social scenes may also provide relevant evidence (for United States parents, Knothe and Walle, 2018).

Our results also suggest that there are no cultural differences in the valence of terms used to describe social events – parents from both countries discussed the negativity as well as the positivity of the negative events but focused solely on the positivity of the positive events. The results are similar to previous studies with Western populations (e.g., Recchia et al., 2014) finding that parents used positive (e.g., good and nice) and negative valence words (e.g., not nice, mean, and bad) for the negative events, but used solely on the positive valence words for the positive events. Other studies (Lagattuta and Wellman, 2002; Recchia et al., 2014) with Western populations also found that conversations about negative personal events were described longer and with more components than conversations about positive personal events. However, for our study, we did not see any differences between positive and negative scenes in the total number of the actor and recipient references for both countries, suggesting that personal events used in previous studies and puppet shows used in this study might elicit different descriptions from parents. This difference between personal events and puppet shows might be also interesting to test in the future.

A previous cross-cultural study (Shimizu et al., 2021) on how children direct visual attention to moral events found that both United States and Japanese preschool-aged children directed gaze more to the recipient than the agent in both positive and negative scenarios. Our study found cross-cultural differences in parents talk in negative social events. A critical next question is how well the talk of parents about social interactions controls the visual attention of children to the participants of social events and how well that visual attention aligns with children’s interpretations of these events.

The results of this study bridge cross-cultural differences previously found in two domains, attention to individuals or relations and differences in the description of social events, particularly focusing on which part(s) of the social event parents discuss. There are cultural differences in how parents talk to their children and these differences are more exaggerated in negative social events. These differences in experiences, small yet pervasive, might create different developmental trajectories to comparable functional ends. A complete theory of a developmental process requires an understanding of how different experiences, such as those observed in this study and the different systems of experiences within culture as a whole interact with fundamental developmental processes to yield cultural differences in both cognitive and social systems.

## Data Availability Statement

The raw data supporting the conclusions of this article will be made available by the authors, without undue reservation.

## Ethics Statement

The studies involving human participants were reviewed and approved by the Indiana University Institutional Review Board. The participants and the participants’ legal guardians provided their written informed consent to participate in this study.

## Author Contributions

MK contributed to all aspects of this research – the conception of the idea, designing, data collection, data coding, analysis, and drafting the article. LS contributed to the conception of the idea, the data analysis, and the drafting of the article. Both authors contributed to the article and approved the submitted version.

## Conflict of Interest

The authors declare that the research was conducted in the absence of any commercial or financial relationships that could be construed as a potential conflict of interest.

## Publisher’s Note

All claims expressed in this article are solely those of the authors and do not necessarily represent those of their affiliated organizations, or those of the publisher, the editors and the reviewers. Any product that may be evaluated in this article, or claim that may be made by its manufacturer, is not guaranteed or endorsed by the publisher.

## Appendix A

The list of puppet shows including the scene description, actor, and recipient of each scene.
